# Perceived Factors That Contributed to Task Termination during Fatiguing Tasks Anchored to Perceptual Intensities

**DOI:** 10.3390/jfmk9030152

**Published:** 2024-08-29

**Authors:** Dolores G. Ortega, Robert W. Smith, Jocelyn E. Arnett, Tyler J. Neltner, Trevor D. Roberts, Richard J. Schmidt, Terry J. Housh

**Affiliations:** 1 Exercise Physiology Laboratory, Department of Nutrition and Health Sciences, University of Nebraska—Lincoln, Lincoln, NE 68510, USA; jarnett4@huskers.unl.edu (J.E.A.); troberts17@huskers.unl.edu (T.D.R.); rschmidt1@unl.edu (R.J.S.); 2 Department of Health, Human Performance, and Sport, Wayne State College, Wayne, NE 68787, USA; bosmith1@wsc.edu; 3 Department of Health and Human Performance, University of Wisconsin—Platteville, Platteville, WI 53818, USA; neltnert@uwplatt.edu

**Keywords:** ratings of perceived exertion, torque, neuromuscular responses, fatigue, effort, motivation, electromyography

## Abstract

This study examined the effects of sustained, isometric forearm flexion tasks anchored to ratings of perceived exertion of 2 (RPE2_FT_) and 8 (RPE8_FT_) on the patterns of fatigue-induced changes in torque and neuromuscular responses, time to task failure (TTF), performance fatigability (% decline in maximal voluntary isometric contraction [MVIC]), and perceived factors that contributed to task termination. Twelve men (mean ± SD: age = 20.9 ± 2.2 yrs) performed MVICs before and after the tasks and completed post-test questionnaires (PTQ). Data were analyzed using polynomial regression analyses, dependent *t*-tests, Spearman’s rank order correlations, and Wilcoxon signed rank tests. The RPE8_FT_ had greater (*p* < 0.001) performance fatigability than the RPE2_FT_, despite no difference (*p* > 0.05) in TTF. During both tasks, there were significant (*p* ≤ 0.05) composite linear decreases for torque, electromyographic amplitude, and neuromuscular efficiency, and substantial individual variability in the neuromuscular responses. There were no significant (*p* > 0.05) associations among the perceived factors and TTF or performance fatigability. Thus, there were RPE-specific differences in performance fatigability, but not TTF or the composite patterns of changes in torque and neuromuscular responses. In addition, in most cases, the individual neuromuscular, but not torque, patterns of responses were RPE-specific. Furthermore, the perceived factors assessed by the PTQ were not related to TTF or performance fatigability.

## 1. Introduction

The study of fatigue has garnered multidisciplinary interest, which has resulted in many different definitions [1] that attempt to characterize its physiological and psychological components [2]. In clinical settings, fatigue is commonly measured as a subjective experience with patients reporting their feelings of “…extreme and persistent tiredness, weakness, or exhaustion that could be mental, physical, or both” [3] (p. 120). With regard to human performance, Enoka and Stuart [1] defined fatigue as an “…acute impairment of performance that includes both an increase in the perceived effort necessary to exert a desired force and an eventual inability to produce this force” (p. 1631). To unify the physiological and psychological aspects of fatigue, Kluger et al. [4] and Enoka and Duchateau [5] have proposed taxonomies that define fatigue as an interaction between performance fatigability (i.e., the objective decline of a performance measure across time) and perceived fatigability (i.e., the changes in the sensations and perceptions associated with performance). Thus, performance fatigability and perceived fatigability are two interdependent attributes of fatigue that are influenced by different factors [4,5]. Specifically, performance fatigability is affected by changes in contractile function and muscle activation, whereas perceived fatigability is affected by changes in homeostasis and the psychological state of the individual [4,5]. While the measures used to assess each attribute of fatigue are dependent on the characteristics of the task [4,5], decreases in maximal voluntary isometric contractions (MVIC) [6,7,8,9,10,11] and self-report scales [7,10,11] are commonly used to examine performance fatigability and perceived fatigability, respectively.

The Omnibus Resistance Exercise 0–10 (OMNI-RES) Scale [12] has been used in recent studies as a self-report scale [7,10,11] as well as a tool to regulate exercise intensity when anchoring fatiguing tasks to a rating of perceived exertion (RPE) [6,8,9,10,11] using the RPE Clamp Model [13]. According to the RPE Clamp Model of Tucker [13], when a fatiguing task is anchored to an RPE value, previous experiences, training status, and the expected duration of the task are used to set the initial intensity at the prescribed RPE. During the fatiguing task, afferent feedback that results from fatigue-induced changes in the physiological systems is interpreted within the brain, which allows for the regulation of exercise intensity and maintenance of the prescribed RPE [13]. A recent study by Smith et al. [10] examined the effects of anchor scheme following sustained, isometric forearm flexion tasks anchored to RPE = 8 and the torque that corresponded to RPE = 8 on performance fatigability (defined as a decrease in torque from pre-test to post-test MVIC) as well as perceived fatigability using a post-test questionnaire (PTQ) that consisted of five items (biceps brachii, forearm muscles, hand muscles, loss of motivation, and loss of focus) rated on a 0 (no contribution) to 5 (greatest level of contribution) scale to determine the location and magnitude of sensations and psychological factors that contributed to the decision to terminate the fatiguing tasks. It was reported that the fatiguing tasks did not result in anchor scheme-specific mean differences in the fatigue-induced decreases in torque or the responses to the PTQ items [10]. Furthermore, in an overlapping sample of 10 subjects, Smith et al. [11] examined the effects of anchoring sustained, isometric forearm flexion tasks anchored to the torque that corresponded to RPE = 2 and RPE = 8 on performance fatigability (defined as a decrease in torque from pre-test to post-test MVIC), RPE at task failure (defined as the time point at which the subjects could no longer maintain the prescribed torque), and perceived fatigability using a PTQ with 10 Likert-type items to determine the perceived sensations and psychological factors that contributed to task termination. It was reported that the fatiguing tasks resulted in no mean differences in performance fatigability, RPE at task failure, or the PTQ item responses, except for pain in the biceps brachii (BB) [11]. Thus, performance fatigability, measured using fatigue-induced decreases in torque from pre-test to post-test MVICs, and perceived fatigability, assessed using PTQs, have been examined following fatiguing tasks anchored to a high RPE value as well as low- and high-intensity target torque values [10,11].

Recent studies have examined the fatigue-induced changes in the amplitude (AMP) of the electromyographic (EMG) signal [6,9,14,15] and neuromuscular efficiency (NME = normalized torque/normalized EMG AMP) [6,9] to make indirect inferences about the motor unit activation strategies used to modulate torque production during fatiguing tasks anchored to a constant RPE. For example, Arnett et al. [6] examined the composite relationships for EMG AMP and NME versus time during sustained, isometric forearm flexion tasks anchored to RPE = 8 at elbow joint angles of 75° and 125° in women. The authors reported linear decreases in EMG AMP and NME during the fatiguing task at the elbow joint angle of 75°, and a quadratic decrease in EMG AMP and a linear decrease in NME during the fatiguing task at the elbow joint angle of 125° [6]. Typically, EMG AMP reflects changes in muscle excitation [16], while NME provides indirect information about the responsiveness of the contractile elements of muscle to neural excitation [17]. Furthermore, decreases in NME have been associated with mechanisms of peripheral fatigue that occur at or distal to the neuromuscular junction [18] due to the accumulation of intramuscular metabolites, including inorganic phosphate (P_i_) and hydrogen ions (H^+^), that result in excitation–contraction coupling failure [19]. Therefore, Arnett et al. [6] suggested that the joint angle-specific composite neuromuscular patterns of responses during fatiguing forearm flexion tasks anchored to a RPE = 8 likely reflected peripheral fatigue.

While recent studies have investigated the effects of joint angle [6] and anchor scheme (RPE versus torque) [9] on the torque and neuromuscular patterns of responses as well as the effect of anchor scheme (RPE versus torque) [10] and a low- versus high-intensity target torque [11] on the perceived sensations and psychological factors that contributed to task termination, no study has examined the effects anchoring fatiguing tasks to a low versus high RPE on the torque and neuromuscular patterns of responses and the perceived factors that contributed to task termination. A recent study [8] with an overlapping sample of 10 subjects examined the pre-test to post-test torque and neuromuscular responses following sustained, isometric forearm flexion tasks anchored to RPE = 2 and RPE = 8, and reported decreases in NME, likely due to peripheral fatigue, but no changes in EMG AMP. It was previously suggested that assessing the time course of fatigue-induced changes in the neuromuscular responses provides additional information about the motor unit activation strategies used to modulate torque production that is not provided from maximal measurements taken prior to and following fatiguing tasks [20]. Furthermore, Keller et al. [14] suggested that the neuromuscular responses during sustained isometric tasks anchored to torque and RPE are reflective of the fatigue-induced physiological changes that underlie torque production and the perception of effort, respectively. Therefore, the purpose of this study was to examine the effects of sustained, isometric forearm flexion tasks anchored to RPE = 2 (RPE2_FT_) and RPE = 8 (RPE8_FT_) on the time-dependent patterns of changes for the composite, inter-, and intra-individual torque and neuromuscular responses as well as time to task failure (TTF), performance fatigability (% decline in MVIC = [((pre-test MVIC − post-test MVIC)/pre-test MVIC) × 100]), and the perceived factors that contributed to task termination. Based on the results of recent studies, it was hypothesized that: (a) there would be no difference in TTF between the fatiguing tasks [8]; (b) the RPE8_FT_ would result in greater performance fatigability than the RPE2_FT_ [8]; (c) there would be differences in the composite patterns of responses for the neuromuscular responses, but not torque [6,9,15]; (d) there would be substantial inter- and intra-individual variability in the patterns of responses for the neuromuscular parameters, but not torque [6,9]; (e) the perceived sensations and psychological factors that contributed to task termination will differ between the fatiguing tasks [11]; and (f) the perceived sensations and psychological factors will not be related to TTF or performance fatigability [11].

## 2. Materials and Methods

### 2.1. Subjects

An a priori sample size calculation (G*Power version3.1.9.4, Düsseldorf, Germany) using previously reported TTF data [10] indicated that a power of 0.95 and an alpha of 0.05 required 7 subjects. Twelve men (mean ± SD: age = 20.9 ± 2.2 yrs.; height = 179.8 ± 5.3 cm; body mass = 80.2 ± 9.9 kg) volunteered to participate in this study. The subjects were recreationally active [21], right hand dominant based on throwing preference [22], and free of upper body pathologies that would affect performance. In addition, the subjects were instructed to avoid upper body exercise at least 24 h prior to testing and avoid consumption of caffeine for at least 6 h prior to testing. The subjects in this study were part of a larger, multiple independent and dependent variable investigation, but none of the data in the present study have been previously published [8,9,11,23]. This study was approved by the Institutional Review Board for Human Subjects (IRB Approval #: 20220521909FB and date of approval: 3 May 2022), and all the subjects signed a written informed consent form and completed a health history questionnaire prior to any testing.

### 2.2. Time Course of Procedures

Each subject visited the laboratory on three separate occasions (orientation session and two test visits), and each visit was separated by at least 24 h. The initial visit was an orientation session where demographic information was recorded, and the subjects were familiarized with the standardized warm-up, testing protocol, the standardized OMNI-RES [12] instructions (Table 1), and the PTQ. Test visits 1 and 2 included the standardized warm-up, pre-test MVIC trials to set a perceptual anchor to RPE = 10, a sustained, isometric forearm flexion task to failure anchored to RPE = 2 (RPE2_FT_) or RPE = 8 (RPE8_FT_) (in randomized order), post-test MVIC trials, and the PTQ. To reflect the approximate point in the range of motion where maximal isometric torque production occurs [24], all forearm flexion muscle actions were performed with the dominant (right) arm at an elbow joint angle of 100°. Furthermore, all forearm flexion muscle actions were performed with the forearm in a neutral position. The EMG signal was recorded from the biceps brachii (BB) during both fatiguing tasks. The time course of procedures is presented in Table 1.

### 2.3. OMNI-RES Scale Standardized Anchoring Procedures

The anchoring instructions used in the present study were originally developed by Gearhart et al. [25] as a standardized method to gauge training intensity during lower body exercise and adapted by Smith et al. [15] for use during isometric forearm flexion tasks anchored to RPE. The following standardized anchoring instructions were read to each subject during the orientation session and prior to the RPE2_FT_ and RPE8_FT_: “You will be asked to set an anchor point for both the lowest and highest values on the perceived exertion scale. To set the lowest anchor, you will be asked to lay quietly without contracting your forearm flexor muscles to familiarize yourself with an RPE of zero. Following this, you will be asked to perform a maximal voluntary isometric contraction to familiarize yourself with an RPE of 10. When instructed to match a perceptual value corresponding to the OMNI-RES scale, perceived exertion should be related to these defined anchors”.

### 2.4. Orientation Session

Each subject’s dominant arm (based on throwing preference), age, height, and body mass were recorded during the orientation session. Then, in accordance with the Cybex II (Cybex II International Inc., Medway, MA, USA) user’s manual, the subjects were oriented to the testing position on an upper body exercise table (UBXT) with the lateral epicondyle of the humerus of the dominant arm aligned with the lever arm of the calibrated isokinetic dynamometer. While positioned on the UBXT, the subjects were familiarized with the PTQ and the OMNI-RES (0–10) RPE scale, which has been shown to be valid and reliable for quantifying perception during resistance exercise [12], and read the standardized OMNI-RES instructions [12,15] used during testing. The subjects then completed the standardized warm-up, 2, 3 s isometric forearm flexion MVICs to set a perceptual anchor corresponding to RPE = 10, and brief sustained, isometric tasks anchored to RPE = 2 and RPE = 8 to become familiar with the anchoring procedures.

### 2.5. Test Visits

During each test visit, subjects were positioned on the UBXT in accordance with the Cybex II user’s manual with the lateral epicondyle of the humerus of the dominant arm aligned with the lever arm of the dynamometer at an elbow joint angle of 100°. The subjects then performed the standardized warm-up followed by 1 min of rest before being read the OMNI-RES instructions relating to the anchoring procedures. For pre-testing, the subjects performed 2, 3 s forearm flexion MVICs and were provided strong verbal encouragement. Following the pre-test MVICs, the subjects performed the sustained, isometric forearm flexion tasks anchored to RPE = 2 (RPE2_FT_) or RPE = 8 (RPE8_FT_) (OMNI-RES scale) at an elbow joint angle of 100°, in a randomized order. To ensure compliance with the prescribed anchors, the subjects were asked their RPE every 30 s during the RPE2_FT_ and every 20 s during the RPE8_FT_. Furthermore, to maintain the appropriate levels of exertion during the fatiguing tasks, the subjects were continuously advised to be attentive and relate the levels of exertion to the previously set anchors of RPE = 0 and RPE = 10. Thus, the subjects were able to adjust their torque to maintain RPE = 2 and RPE = 8 as fatigue developed. The fatiguing tasks were sustained to task failure, which was defined as torque reduced to zero. At task failure, the RPE2_FT_ and RPE8_FT_ were terminated, TTF was recorded, 2, 3 s post-test MVICs were performed in a manner identical to the pre-test MVICs, and the PTQ was completed.

### 2.6. Post-Test Questionnaire

During the orientation session and following the post-test MVICs during test visits 1 and 2, the PTQ was explained to the subjects. The PTQ was used to identify the site of perceived sensations and psychological factors and determine their contribution to the subjects’ decision to terminate the tasks. The PTQ included 10 Likert-type items that were divided into 5 subcategories, which included (1) BB; (2) forearm muscles [FM]; (3) hand muscles [HM]; (4) focus; and (5) motivation. The 10 Likert-type items included (1) BB fatigue; (2) BB pain; (3) FM fatigue; (4) FM pain; (5) HM fatigue; (6) HM pain; (7) loss of focus [LOF]; (8) Motivation 1 [i.e., the subject expended the maximal effort they could give, which resulted in the decision to terminate the task]; (9) Motivation 2 [i.e., the subject perceived that the task could not be accomplished, and decided to terminate the task]; and (10) Motivation 3 [i.e., the subject became bored and decided to terminate the task]. Each of the Likert-type items was rated on a 5-point (1–5) Likert-type scale [26,27,28] with definitions associated with the following numbers: 1, “Strongly disagree”; 2, “Disagree”; 3, ‘”Neither agree nor disagree”; 4, “Agree”; and 5, “Strongly agree”. The primary (BB) and synergistic (FM and HM) muscle groups were selected to identify the potential sites of muscle fatigue and pain (i.e., perceived sensations) that subjects perceived to have contributed to the decision to terminate the task. In the present study, the feelings of fatigue and pain were defined, respectively, as the inability of the muscles to produce sufficient torque [29] and “The intensity of hurt you are feeling” [30] (p. 1011). Loss of focus was used to assess if attention shifted away from the fatiguing tasks to other tasks, responsibilities, and/or sensations, which resulted in the inability to maintain the prescribed RPE, and, thus, contributed to task termination. Motivation 1 and 2 were adapted from the Motivation Intensity Theory described by Brehm and Self [31] to assess the potential cause of task termination following the fatiguing tasks.

### 2.7. Electromyographic and Torque Acquisition

In accordance with the recommendations of the Surface Electromyography for the Non-Invasive Assessment of Muscles [32], pre-gelled surface EMG electrodes (Ag/AgCl, Accusensor; Lynn Medical, Wixom, MI, USA) were placed in a bipolar arrangement (30-mm center-to-center) on the BB of the dominant arm during each test visit. The electrodes were placed over the BB between the medial acromion process and the antecubital fossa, at one-third the distance from the antecubital fossa, and the reference electrode was placed on the radial styloid process of the forearm. Prior to electrode positioning, the skin was carefully shaved, abraded, and cleaned with alcohol.

The raw EMG signal was digitized at 2000 samples per second using a 12-bit analog-to-digital converter (Model MP150; Biopac Systems, Inc., Goleta, CA, USA). The EMG signals were then stored on a personal computer (Dell Inspiron Dell Inc., Round Rock, TX, USA) for signal processing that was performed using custom programs written with LabVIEW programming software (version 22.3f0, National Instruments, Austin, TX, USA). The EMG signals were digitally band-pass filtered (fourth-order Butterworth) at 10–500 Hz. The TTF (0–100%) was divided into 10% increments and a 1 s epoch from the center of each 10% increment (i.e., 500 ms before and 500 ms after) was used to calculate the AMP (root mean square [μVrms]) for the EMG signal. Neuromuscular efficiency was calculated by dividing the normalized torque by the normalized EMG AMP [17,33]. The torque signals were sampled from the digital torque of the calibrated Cybex II isokinetic dynamometer and stored on a personal computer (Dell Inspiron Dell Inc., Round Rock, TX, USA) for analysis.

### 2.8. Statistical Analysis

The test–retest reliability for the pre-test MVIC, EMG AMP, and NME values at RPE = 2 and RPE = 8 (visit 1 vs. visit 2) were assessed with repeated measures ANOVAs to evaluate systematic error and a 2,1 model was used to determine intraclass correlation coefficients (ICC) [34]. The corresponding pre-test forearm flexion MVIC with the greatest torque production was used to normalize the torque, EMG AMP, and NME for each 10% of the TTF for the RPE2_FT_ and RPE8_FT_. Separate polynomial regression analyses (linear and quadratic) were used to define the individual and composite relationships for the normalized torque (10–100% TTF), EMG AMP (10–100% TTF), and NME (10–90% TTF) values versus time during the RPE2_FT_ and RPE8_FT_. Dependent *t*-tests were used to determine mean differences for TTF and performance fatigability (% decline in MVIC = [((pre-test MVIC − post-test MVIC)/pre-test MVIC) × 100]) between the fatiguing tasks, and effect sizes were reported as Cohen’s *d*. Spearman’s rank order correlations [35] were performed to assess the associations among the 10 Likert-type items from the PTQ, TTF, and performance fatigability from the RPE2_FT_ and RPE8_FT_, and zero-order correlations were reported as r_s_. In addition, the coefficient of determination, reported as r_s_^2^, was calculated for zero-order correlations to estimate the amount of shared variance between variables. Wilcoxon signed rank [36] test was used to determine the mean differences between the RPE2_FT_ and RPE8_FT_ for the average values from the 10 Likert-type items from the PTQ. A *p*-value ≤ 0.05 was considered statistically significant for the analyses. All the statistical analyses were completed in IBM SPSS v. 29 (Armonk, NY, USA).

## 3. Results

### 3.1. Reliability

Table 2 includes the test–retest reliability parameters (*p*-value (systematic error), ICC, and ICC_95%_) for MVIC, EMG AMP, and NME. There were no mean differences for test versus retest for MVIC (*p* = 0.379, ICC = 0.902) or NME (*p* = 0.088, ICC = 0.903); however, there was a significant (*p* = 0.020, ICC = 0.892) difference for EMG AMP.

### 3.2. Time to Task Failure and Performance Fatigability

**Figure 1:**
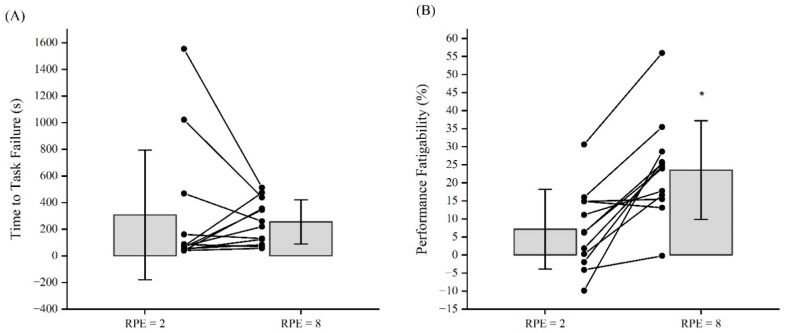
(**A**) Mean (±SD) time to task failure (TTF) values for the sustained, isometric forearm flexion tasks anchored to RPE = 2 (RPE2_FT_) and RPE = 8 (RPE8_FT_). (**B**) Mean (±SD) performance fatigability (% decline in MVIC = [((pre-test MVIC − post-test MVIC)/pre-test MVIC) × 100]) values for the sustained, isometric forearm flexion tasks anchored to RPE = 2 (RPE2_FT_) and RPE = 8 (RPE8_FT_). Spaghetti graphs are the individual subject responses. * RPE = 8 > RPE = 2 (*p* < 0.001, *d* = −1.359).

The results of the dependent *t*-test for TTF indicated no significant (*p* = 0.668, *d* = 0.127) mean difference between the RPE2_FT_ (306.9 ± 486.4 s) and RPE8_FT_ (255.5 ± 166.3 s) (Figure 1). The results of the dependent *t*-test for performance fatigability indicated a significant (*p* < 0.001, *d* = −1.359) mean difference between the RPE2_FT_ (7.2 ± 11.0%) and RPE8_FT_ (23.5 ± 13.7%) (Figure 1).

### 3.3. Torque Responses

**Figure 2:**
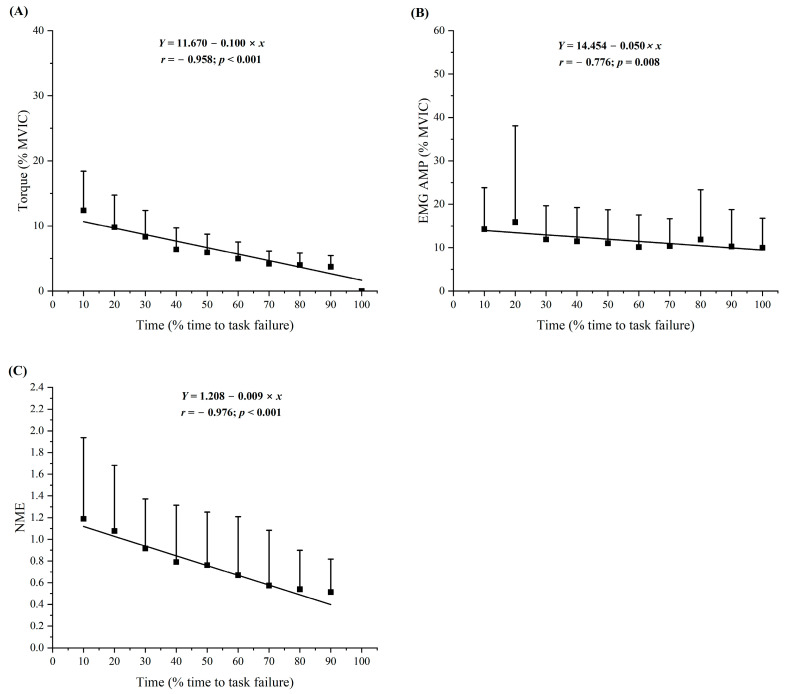
Time course of changes (mean ± SD) for the normalized (% of pre-test MVIC) torque and neuromuscular values for the sustained, isometric forearm flexion task anchored to RPE = 2 (RPE2_FT_) at an elbow joint angle of 100°. Regression analyses represent torque and electromyographic amplitude values from 10–100% time to task failure and neuromuscular efficiency values from 10–90% time to task failure. (**A**) Torque, (**B**) electromyographic amplitude (EMG AMP), and (**C**) neuromuscular efficiency (NME).

During the RPE2_FT_, the normalized individual and composite torque responses indicated significant negative linear relationships for torque vs. time (r = −0.770 to −0.978) for 12 of the 12 subjects, and a negative linear relationship (r = −0.958) for the composite data (Figure 2 and Table 3).

**Figure 3:**
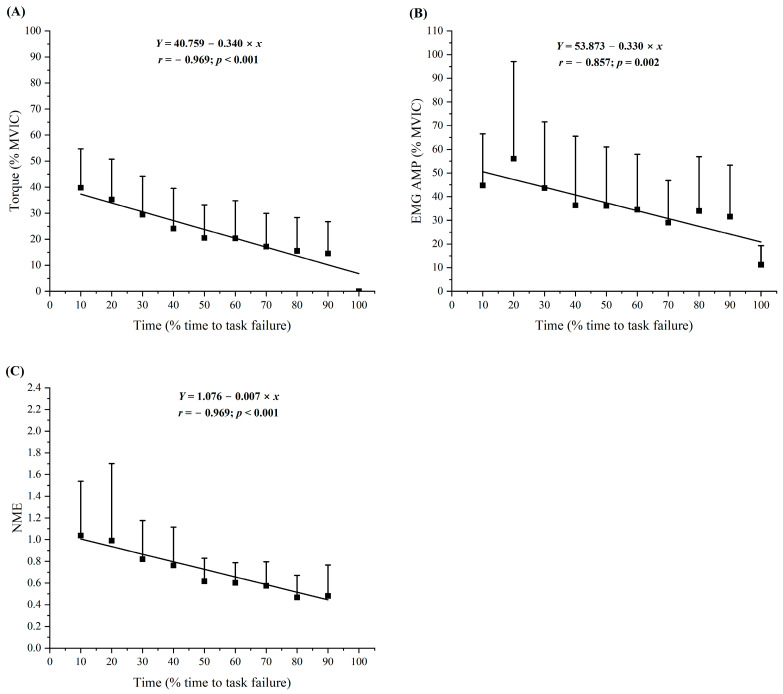
Time course of changes (mean ± SD) for the normalized (% of pre-test MVIC) torque and neuromuscular values for the sustained, isometric forearm flexion task anchored to RPE = 8 (RPE8_FT_) at an elbow joint angle of 100°. Regression analyses represent torque and electromyographic amplitude values from 10–100% time to task failure and neuromuscular efficiency values from 10–90% time to task failure. (**A**) Torque, (**B**) electromyographic amplitude (EMG AMP), and (**C**) neuromuscular efficiency (NME).

During the RPE8_FT_, the normalized individual and composite torque responses indicated significant negative linear relationships for torque vs. time (r = −0.761 to −0.947) for 9 of the 12 subjects, negative quadratic relationships (R = −0.933 to −0.969) for 3 subjects, and a negative linear relationship (r = −0.969) for the composite data (Figure 3 and Table 4).

### 3.4. Electromyographic Amplitude Responses

During the RPE2_FT_, the normalized individual and composite EMG AMP responses indicated significant negative linear relationships for EMG AMP vs. time (r = −0.723 to −0.930) for 7 of the 12 subjects, no relationships for 5 subjects, and a negative linear relationship (r = −0.776) for the composite data (Figure 2 and Table 3).

During the RPE8_FT_, the normalized individual and composite EMG AMP responses indicated significant negative linear relationships for EMG AMP vs. time (r = −0.635 to −0.947) for 5 of the 12 subjects, negative quadratic relationships (R = −0.436 to −0.880) for 3 subjects, no significant relationships for 4 subjects, and a negative linear relationship (r = −0.857) for the composite data (Figure 3 and Table 4).

### 3.5. Neuromuscular Efficiency Responses

During the RPE2_FT_, the normalized individual and composite NME responses indicated significant negative linear relationships for NME vs. time (r = −0.704 to −0.955) for 10 of the 12 subjects, no relationships for 2 subjects, and a negative linear relationship (r = −0.976) for the composite data (Figure 2 and Table 3).

During the RPE8_FT_, the normalized individual and composite NME responses indicated significant negative linear relationships for NME vs. time (r = −0.699 to −0.946) for 7 of the 12 subjects, a negative quadratic relationship (R = −0.408) for 1 subject, no significant relationships for 4 subjects, and a negative linear relationship (r = −0.969) for the composite data (Figure 3 and Table 4).

### 3.6. Relationships among Perceived Sensations, Psychological Factors, Time to Task Failure, and Performance Fatigability

The zero-order correlations for the 10 Likert-type items, TTF, and performance fatigability for the RPE2_FT_ and RPE8_FT_ are presented in Table 5 and Table 6, respectively. For the Likert-type items from the PTQ following the RPE2_FT_, the Spearman’s rank order correlations indicated significant (*p* ≤ 0.05) associations for BB fatigue vs. BB pain (r_s_ = 0.663), BB fatigue vs. FM fatigue (r_s_ = 0.694), BB fatigue vs. HM fatigue (r_s_ = 0.654), BB pain vs. FM pain (r_s_ = 0.675), BB pain vs. HM fatigue (r_s_ = 0.720), BB pain vs. HM pain (r_s_ = 0.648), FM fatigue vs. FM pain (r_s_ = 0.788), FM fatigue vs. HM fatigue (r_s_ = 0.763), FM fatigue vs. HM pain (r_s_ = 0.617), FM pain vs. HM fatigue (r_s_ = 0.689), FM pain vs. HM pain (r_s_ = 0.585), HM fatigue vs. HM pain (r_s_ = 0.942), LOF vs. Motivation 2 (r_s_ = 0.703), LOF vs. Motivation 3 (r_s_ = 0.691), and Motivation 2 vs. Motivation 3 (r_s_ = 0.822). There were no significant (*p* > 0.05, r_s_ = −0.263 to 0.458) associations for the perceived sensations and psychological factor items versus TTF or performance fatigability. For the Likert-type items from the PTQ following the RPE8_FT_, the Spearman’s rank order correlations indicated significant (*p* ≤ 0.05) associations for FM fatigue vs. FM pain (r_s_ = 0.904), FM fatigue vs. HM fatigue (r_s_ = 0.726), FM fatigue vs. HM pain (r_s_ = 0.679), FM pain vs. HM fatigue (r_s_ = 0.630), FM pain vs. HM pain (r_s_ = 0.632), FM pain vs. Motivation 3 (r_s_ = 0.611), HM fatigue vs. HM pain (r_s_ = 0.856), and LOF vs. Motivation 2 (r_s_ = 0.690). There were no significant (*p* > 0.05, r_s_ = −0.315 to 0.547) associations for the perceived sensations and psychological factor items versus TTF or performance fatigability.

### 3.7. Mean Differences between RPE2_FT_ and RPE8_FT_ for Perceived Sensations and Psychological Factors

Ten separate Wilcoxon signed rank tests were used to examine the average differences between the RPE2_FT_ and RPE8_FT_ for the 10 Likert-type item scores from the PTQ. There were significant differences between the RPE2_FT_ and RPE8_FT_ item scores for BB fatigue (2.3 ± 1.4 vs. 4.0 ± 1.3, Z = −2.687, *p* = 0.007) and BB pain (1.8 ± 0.9 vs. 3.8 ± 1.3, Z = −2.965, *p* = 0.003). There were no significant differences, however, between the RPE2_FT_ and RPE8_FT_ items scores for FM fatigue (2.1 ± 1.1 vs. 2.5 ± 1.1, Z = −1.414, *p* = 0.157), FM pain (2.0 ± 1.0 vs. 2.5 ± 1.1, Z = −1.730, *p* = 0.084), HM fatigue (3.1 ± 1.6 vs. 3.0 ± 1.4, Z = −0.184, *p* = 0.854), HM pain (2.8 ± 1.4 vs. 2.7 ± 1.2, Z = −0.649, *p* = 0.516), LOF (2.1 ± 1.1 vs. 1.6 ± 0.8, Z = −1.730, *p* = 0.084), Motivation 1 (3.5 ± 1.2 vs. 3.8 ± 1.5, Z = −0.954, *p* = 0.340), Motivation 2 (1.7 ± 0.8 vs. 1.6 ± 0.5, Z = −0.577, *p* = 0.564), or Motivation 3 (1.5 ± 0.8 vs. 1.2 ± 0.4, Z = −1.633, *p* = 0.102). The individual subject scores for each item from the PTQ following the RPE2_FT_ and RPE8_FT_ are provided in Table 7 and Table 8, respectively. The composite scores from the PTQ from the RPE2_FT_ and RPE8_FT_ are provided in Table 9.

## 4. Discussion

The test–retest reliability analyses for MVIC, EMG AMP, and NME in the present study are presented in Table 2. There were no significant mean differences for test versus retest reliability for forearm flexion MVIC and NME, and the ICC values (R = 0.902 and R = 0.903, respectively) reflected excellent reliability [37]. The ICC reported for MVIC in the present study was similar to that reported (R = 0.928) by Smith et al. [38] for forearm flexion MVIC at an elbow joint angle of 100° in men. For EMG AMP in the present study, however, there was a significant difference between the mean values for test versus retest reliability, but the ICC (R = 0.892) reflected excellent reliability [37]. The ICC reported for EMG AMP in the present study was higher than that reported (R = 0.610) by Smith et al. [38] for forearm flexion MVIC at an elbow joint angle of 100° in men. It has been suggested that ICCs can be affected by the degree of variability in the sample [39] whereas slight day-to-day changes in the location of the electrodes used to record the EMG signal can result in variations in the absolute values of the neuromuscular parameters used in the test–retest reliability analyses [40].

In the present study, there was no significant difference in TTF between the RPE2_FT_ (306.9 ± 486.4 s) and RPE8_FT_ (255.5 ± 166.3 s). This finding was similar to that of our recent study [8] that utilized an overlapping sample of 10 men and found no difference in TTF following sustained, isometric forearm flexion tasks anchored to RPE = 2 (332.3 ± 560.0 s) and RPE = 8 (273.0 ± 188.5 s). The ability to reduce torque throughout a fatiguing task anchored to RPE, compared to the increase in RPE when a fatiguing task is anchored to torque [41], affects the interactions among perceived fatigability, performance fatigability, and TTF. When fatiguing tasks are anchored to torque, there is an inverse relationship between the relative intensity of the torque at which the task is sustained and TTF [11,12,42,43]. The results of the present study indicated, however, that when the tasks were anchored to perceptual intensities, the initial torque values at the beginning of the tasks (RPE2_FT_ = 17.0 ± 5.6 Nm vs. RPE8_FT_ = 51.2 ± 13.5 Nm) did not affect the TTF and that self-determined differences in the rates of decrease in torque based on the perception of exertion throughout the tasks (RPE2_FT_ = 0.06 Nm∙s^−1^ vs. RPE8_FT_ = 0.20 Nm∙s^−1^) resulted in no mean differences in TTF between the tasks. Future studies should further examine these findings in different muscle groups, at various levels of perceptual intensities, and during dynamic as well as isometric muscle actions.

In the present study, the RPE8_FT_ (23.5 ± 13.7%) resulted in greater performance fatigability than the RPE2_FT_ (7.2 ± 11.0%), despite the lack of difference in TTF. These findings were consistent with those of Ortega et al. [8] in an overlapping sample of 10 men that reported greater performance fatigability following a sustained, isometric forearm flexion task anchored to RPE = 8 (26.2%) than RPE = 2 (11.6%) with no difference in TFF between the sustained tasks (RPE = 2: 332.3 ± 560.0 s vs. RPE = 8: 273.0 ± 188.5 s). The results of the present study, however, were not consistent with those of Smith et al. [11], who utilized an overlapping sample of 10 men and reported no difference in the decline in pre-test to post-test MVIC torque (i.e., performance fatigability) following sustained, isometric forearm flexion tasks anchored to the torque that corresponded to RPE = 2 (pre-test MVIC torque = 97.6 ± 20.7 vs. post-test MVIC torque = 68.8 ± 21.4 Nm) and RPE = 8 (pre-test MVIC torque = 92.7 ± 20.8 vs. post-test MVIC torque = 69.1 ± 12.7 Nm), despite a difference in TTF (fatiguing task anchored to the torque that corresponded to RPE = 2: 245.0 ± 177.0 s vs. fatiguing task anchored to the torque that corresponded to RPE = 8: 36.8 ± 11.1 s). Thus, the present findings in conjunction with those of Ortega et al. [8] and Smith et al. [11] have shown that fatiguing tasks anchored to RPE differ from those anchored to torque with regard to the effects of exercise intensity on TTF and performance fatigability. That is, when the tasks were anchored to RPE = 2 and RPE = 8, there were differences in performance fatigability, but not TTF. When tasks were anchored to the torque at RPE = 2 and RPE = 8, however, there was a difference in TTF, but not performance fatigability. Smith et al. [11] suggested that the similarity in performance fatigability between the two fatiguing tasks anchored to torque was due to the small muscle mass being engaged [44] and the attainment of the sensory tolerance limit (STL) [45]. According to Thomas et al. [44], the magnitude of performance fatigability is dependent on the mode and intensity of a fatiguing task. Specifically, tasks that activate a larger amount of muscle mass and/or are performed at an exercise intensity above the critical intensity result in greater performance fatigability due to disruptions to whole-body homeostasis than tasks which activate less muscle mass and are performed below the critical intensity [44]. The STL theory, proposed by Gandevia [18] and conceptualized by Hureau et al. [45], suggests that global sensory feedback from primary and remote muscles as well as feedforward corollary discharges associated with central motor command are summated and limit exercise tolerance once a finite level of stimulation is reached. Perhaps, when anchored to RPE, the conscious decisions to decrease torque at rates related to the initial intensities of the muscle actions is designed to maintain the constant perceptual intensity and allow the tolerable continuation of the task without reaching the STL [18,45]. Therefore, reaching a torque of zero and task termination occurs based on the requirement to maintain a constant perceptual intensity and not limitations associated with the STL. Furthermore, in the present study, the lower initial torque at the beginning of the RPE2_FT_ (17.0 ± 5.6 Nm) than the RPE8_FT_ (51.2 ± 13.5 Nm) required more fine-tuned decreases in torque to avoid reaching a torque of zero before the STL was attained. Thus, the difference in performance fatigability during the RPE2_FT_ and RPE8_FT_ was likely due to the difference in the initial torque values, the rate of torque decreases, and the ability to maintain the perception of the task without reaching the STL.

In the present study, the composite patterns of responses for torque indicated linear decreases for both the RPE2_FT_ and RPE8_FT_. Typically, during fatiguing tasks anchored to RPE, the ability to adjust torque to maintain the perceptual intensity results in torque decreases [6,9,14,15,46]. The patterns of responses (linear vs. quadratic) for torque, however, have differed between studies [6,9,14,15,46]. For example, linear decreases in torque were reported during sustained, isometric forearm flexion tasks anchored to RPE = 7 at an elbow joint angle of 100° in women [15] as well as RPE = 4 in men [9]. Conversely, Arnett et al. [6] reported quadratic decreases in torque following sustained, isometric forearm flexion tasks anchored to RPE = 8 at elbow joint angles of 75° and 125° in women. Quadratic decreases in torque were also reported during sustained, isometric leg extension tasks anchored to RPE = 5 in men [46] and women [14]. Thus, the RPE value used to anchor a fatiguing task may affect the pattern, but not the direction, of the torque response. In addition, the pattern of response may also be dependent on the muscle group of interest and/or the joint angle at which the fatiguing task was performed.

The composite patterns of responses for normalized EMG AMP and NME indicated linear decreases for both the RPE2_FT_ (Figure 2) and RPE8_FT_ (Figure 3). Recent studies have also reported linear [6,9] or quadratic [6,15] decreases in EMG AMP as well as linear decreases in NME [6,9] during sustained, isometric forearm flexion tasks anchored to RPE. Typically, EMG AMP reflects muscle excitation [16] and can be used to calculate NME (normalized torque/normalized EMG AMP) [33]. The NME provides an indirect estimation of the response of the contractile elements of muscle to neural excitation [17]. Decreases in NME generally occur when there is a disproportionate decrease in normalized MVIC torque compared to normalized EMG AMP [33] and are indicative of peripheral mechanisms of fatigue. Peripheral fatigue, which includes impairments in excitation–contraction coupling [47], occurs at or distal to the neuromuscular junction [18] as a result of the accumulation of intramuscular metabolites (primarily P_i_ and secondarily H^+^) [19]. Alternatively, the accumulation of H^+^ in the interstitial space sensed by group III/IV afferent neuron can result in central fatigue [19]. Specifically, central fatigue occurs proximal to the neuromuscular junction [18] and is associated with decreases in neural drive, motor neuron responsiveness to synaptic input, and force production [48]. Thus, the decrease in NME during the RPE2_FT_ and RPE8_FT_ suggested the presence of peripheral fatigue that resulted in excitation–contraction coupling failure, while the decrease in EMG AMP suggested a decrease in muscle excitation. Future studies should use the interpolated twitch and resting potentiated twitch amplitude techniques [49,50] to differentiate the central versus peripheral mechanisms of fatigue affecting the neuromuscular responses and modulating torque production.

In the present study, 100% of the individual patterns of responses for torque during the RPE2_FT_ and 75% during the RPE8_FT_ matched the composite patterns of responses (Table 3 and Table 4). The percentage (75–100%) of inter-individual responses that matched the composite responses were similar to those reported (100%) for sustained forearm flexion tasks anchored to RPE = 4 at an elbow joint angle of 100° in men [9], RPE = 7 (90.9%) at an elbow joint angle of 100° in women [15], and RPE = 8 (55.6–77.8%) at elbow joint angles of 75° and 125° in women [6]. Arnett et al. [6] also reported that seven of the nine (77.8%) subjects had the same individual torque responses during the fatiguing tasks performed at elbow joint angles of 75° and 125°. In the present study, 9 of the 12 (75%) subjects demonstrated the same torque patterns of responses for the RPE2_FT_ and RPE8_FT_. Thus, the inter-individual and intra-individual patterns of responses for torque suggested that in most cases, but not all, the RPE values used to anchor the fatiguing tasks did not affect the individual responses.

For the RPE2_FT_, 58.3% (7 of 12) and 83.3% (10 of 12) of the individual responses for EMG AMP and NME, respectively, matched the composite patterns of responses (Table 3). For the RPE8_FT_, 41.7% (5 of 12) of the individual responses for EMG AMP and 58.3% (7 of 12) for NME matched the composite patterns of responses (Table 4). These findings were consistent with those of recent studies that examined the effects of joint angle [6] and anchor scheme (RPE vs. torque) [9] on inter- and intra-individual neuromuscular responses during fatiguing forearm flexion tasks. Specifically, Arnett et al. [6] reported that 77.8% of individual responses for EMG AMP during a sustained, isometric forearm flexion task anchored to RPE = 8 at an elbow joint angle of 75° matched the direction (but not the pattern) of the composite response, while 66.6% of the individual responses at an elbow joint angle of 125° matched the composite response. For NME, 100% of the individual responses matched the direction of the composite responses for the fatiguing tasks performed at elbow joint angles of 75° and 125° [6]. Ortega et al. [9] reported that 50% of the inter-individual responses for EMG AMP matched the composite patterns of responses following sustained, isometric forearm flexion tasks anchored RPE = 4 and the torque that corresponded to RPE = 4. For NME, 83.3% of the individual responses matched the composite pattern of response for the fatiguing task anchored to RPE, while 100% of the individual responses matched the composite response for the fatiguing task anchored to torque [9]. Thus, the present findings in conjunction with those of Arnett et al. [6] and Ortega et al. [9] suggested that the amount of muscle excitation used to modulate torque production and maintain the perceptual intensity during the fatiguing tasks as well as the NME that resulted from the adjustments in torque and EMG AMP varied on a subject-to-subject basis. With regard to the intra-individual differences, Arnett et al. [6] indicated that 55.6% of the individual responses for EMG AMP and 66.7% for NME varied between joint angles. Ortega et al. [9] reported that 50% of the individual responses for EMG AMP and 83.3% for NME demonstrated the same patterns of responses between the fatiguing tasks anchored to RPE = 4 and the torque that corresponded to RPE = 4 [9]. In the present study, 3 of the 12 (25%) and 6 of the 12 (50%) subjects demonstrated the same intra-individual patterns of responses for EMG AMP and NME, respectively, for the RPE2_FT_ and RPE8_FT_ (Table 3 and Table 4). Therefore, while recent studies suggested that individual neuromuscular responses may be dependent on joint angle [6] and anchor scheme [9], the intra-individual responses in the present study indicated that subjects may modulate torque production using different motor unit activation strategies during fatiguing tasks anchored to a low versus high perceptual intensity. Furthermore, the present findings supported the recommendations of previous studies [6,9,15] that, in addition to composite responses, neuromuscular responses should be assessed on a subject-to-subject basis due to inter- and intra-individual variability.

To identify and determine whether the perceived sensations (i.e., fatigue and pain from the BB, FM, and HM) and psychological factors (i.e., LOF and motivation) contributed to the decision to terminate the fatiguing tasks, subjects completed PTQs after the RPE2_FT_ and RPE8_FT_. The present findings indicated mean differences in the responses for BB fatigue (RPE2_FT_: 2.3 ± 1.4 vs. RPE8_FT_: 4.0 ± 1.3) and BB pain (RPE2_FT_: 1.8 ± 0.9 vs. RPE8_FT_: 3.8 ± 1.3), but not for any of the other PTQ items (Table 7 and Table 8). In an overlapping sample of 10 subjects, Smith et al. [11] used the same PTQ as that in the present study to examine the perceived sensations and psychological factors that contributed to the decision to terminate sustained, isometric forearm flexion tasks anchored to the torque (not RPE) that corresponded to RPE = 2 and RPE = 8. The present findings were in partial agreement to those reported by Smith et al. [11], which indicated a mean difference in BB pain following fatiguing tasks anchored to the torque that corresponded to RPE = 2 (3.7 ± 1.3) and RPE = 8 (2.8 ± 1.2), but no mean differences in BB, FM, and HM fatigue; FM and HM pain; LOF; or Motivation 1, Motivation 2, and Motivation 3. In addition to the difference in the mean response for BB pain, Smith et al. [11] also indicated that there were individual differences in the PTQ item responses. Specifically, it was reported that BB fatigue and Motivation 1 were the predominant contributors to task termination for the fatiguing tasks anchored to torque because 90% of the subjects (18 of 20) selected them on the PTQs; however, they were not the only contributors. For instance, 10–20% of the subjects indicated that both fatigue and pain from the synergistic muscles (FM and HM) also contributed to the decision to terminate the fatiguing tasks [11]. In the present study, there were individual differences in the PTQ item responses as well. For example, for the RPE2_FT_, HM fatigue (59% of the sample agreed or strongly agreed) and Motivation 1 (50% of the sample agreed or strongly agreed) were the predominant sensations and psychological factors that contributed to the decision to terminate the task (Table 9). In contrast, for the RPE8_FT_, BB fatigue (84% of the sample agreed or strongly agreed), BB pain (75% of the sample agreed or strongly agreed), and Motivation 1 (75% of the sample agreed or strongly agreed) were the predominant sensations and psychological factors that contributed to task termination (Table 9). None of the subjects, however, reported that Motivation 2 or Motivation 3 contributed to the decision to terminate either of the fatiguing tasks. For LOF, only subject 10 for the RPE8_FT_ (Table 8) and subject 7 for the RPE2_FT_ (Table 7) reported that it contributed to the decision to terminate the task. Subject 10 also reported that Motivation 1 influenced the decision to terminate the RPE2_FT_ but not the RPE8_FT_, while subject 11 reported that Motivation 1 influenced the decision to terminate the RPE8_FT_ but not the RPE2_FT_ (Table 7 and Table 8). Thus, the results of the present study indicated that the predominant perceived sensations and psychological factors that contributed to task termination were dependent on the RPE value used to anchor the fatiguing tasks. Furthermore, for each subject, the decision to terminate the fatiguing tasks was influenced by various PTQ items, rather than one single item; therefore, individual variability should be considered when examining factors that contribute to task termination.

Previously, in an overlapping sample of 10 subjects, Smith et al. [11] examined the relationships among the perceived sensations, psychological factors, TTF, and performance fatigability for sustained, isometric forearm flexion tasks anchored to the torque (not RPE) that corresponded to RPE = 2 and RPE = 8. Shared common variances of 41.6–89.9% were reported among the perceived sensations and psychological factors examined using the same PTQ as that in the present study, but no relationships were reported between the PTQ items and TTF or performance fatigability [11]. It was suggested that the lack of associations between the PTQ items and the objective measures of fatigue (i.e., TTF and performance fatigability) at task failure was due to “…an unwillingness to continue the task and not an inability to produce sufficient torque” [11] (p. 10) since the subjects produced post-test MVIC torque values (average torque produced after lower intensity task: 68.8 ± 21.4 Nm; average torque produced after higher intensity task: 69.1 ± 12.7 Nm) that were greater than the target torque for the fatiguing tasks (average target torque for the lower intensity task: 23.8 ± 7.1 Nm; average target torque for the higher intensity task: 60.9 ± 11.4 Nm). Furthermore, Smith et al. [11] hypothesized that the disassociation between the PTQ items and the measures of fatigue suggested that the decisions to terminate the fatiguing tasks may have been influenced by factors not examined using the PTQ, such as discomfort [51], effort [51,52], and affective valence [53]. In the present study, the perceived sensations and psychological factors shared 34.2–88.7% and 37.3–81.7% common variance for the RPE2_FT_ and RPE8_FT_, respectively (Table 5 and Table 6). None of the PTQ items, however, were related to TTF or performance fatigability for either of the fatiguing tasks (Table 5 and Table 6). Thus, the findings of the present study and those of Smith et al. [11] indicated that the perceived sensations and psychological factors assessed by the PTQ were not related to TTF or performance fatigability for fatiguing tasks anchored to torque or RPE. In addition, similar to the results of Smith et al. [11], the dissociation between the PTQ items and the measures of fatigue suggested that the decisions to terminate the RPE2_FT_ and RPE8_FT_ may have been influenced by factors not examined in PTQ of the present study.

A limitation of the present study was that only EMG AMP and NME of the BB were used to make inferences about the motor unit activation strategies modulating torque production during the RPE2_FT_ and RPE8_FT_. Future studies should simultaneously assess neuromuscular responses from the BB, brachioradialis, and brachialis muscles to provide a more complete understanding of the fatigue-induced changes in motor unit activation strategies during forearm flexion tasks anchored to RPE and torque. In addition, future studies should compare the neuromuscular responses for isometric versus dynamic muscle actions during tasks anchored to perceptual intensities. The PTQ in the present study examined the contribution of fatigue and pain of the primary and synergist muscles involved during forearm flexion muscle actions as well as loss of focus and motivation on the decision to terminate the fatiguing tasks. Future studies should use PTQs that include items that assess other factors that contribute to perceived fatigability. Furthermore, the results of the present study are limited to recreationally active college-aged men, and, therefore, should be replicated using a larger sample size that includes women and individuals with different levels of physical activity.

## 5. Conclusions

In conclusion, due to the unique characteristics associated with tasks anchored to RPE, the RPE8_FT_ resulted in greater performance fatigability than the RPE2_FT_, despite no difference in TTF. Furthermore, during both fatiguing tasks, there were composite decreases in torque and EMG AMP that were likely related to the ability to decrease torque to maintain the perceptual intensities, as well as decreases in NME, which suggested the presence of peripheral mechanisms of fatigue. The inter- and intra-individual variability in the torque and neuromuscular patterns of responses suggested that in most cases, but not all, the RPE value used to anchor the fatiguing tasks affected the neuromuscular patterns of responses, but not the torque patterns of responses. In addition, except for BB fatigue and BB pain, there were no mean differences in the PTQ responses between the RPE2_FT_ and RPE8_FT_. There were, however, individual differences that suggested that multiple, not just one, PTQ items contributed to task termination. There were also intercorrelations between the perceived sensations and psychological factors assessed by the PTQs, however, the PTQ items were not related to TTF or performance fatigability. Thus, the present findings indicated that performance fatigability, but not TTF or the composite patterns of responses for torque, EMG AMP, and NME, was dependent on the RPE value used to anchor the fatiguing tasks. Furthermore, the decisions to terminate the RPE2_FT_ and RPE8_FT_ were related to multiple perceived sensations and psychological factors; however, the perceived factors assessed by the PTQ were not related to TTF or performance fatigability.

## Figures and Tables

**Table 1:** The time course of procedures.

Orientation Session	Test Visits 1 and 2
Informed consent. Health history questionnaire. Age, height, and body mass recorded. Familiarized to testing procedures. Read the standardized anchoring instructions (OMNI-RES scale). Standardized warm-up: 4, 3 s submaximal (50–75% max effort) isometric forearm flexion muscle actions. 2, 3 s isometric forearm flexion MVICs to set a perceptual anchor of RPE = 10. Brief sustained, isometric forearm flexion tasks anchored to RPE = 2 and RPE = 8.	Standardized warm-up. Read the standardized anchoring instructions (OMNI-RES scale). Pre-test: 2, 3 s MVICs at an elbow joint angle of 100°. Sustained, isometric forearm flexion task anchored to RPE = 2 or RPE = 8 (OMNI-RES scale) performed to task failure at an elbow joint angle of 100°, in a randomized order. Post-test: 2, 3 s MVICs at an elbow joint angle of 100° and post-test questionnaire.

OMNI-Res Scale = Omnibus resistance exercise scale; MVIC = maximal voluntary isometric contraction; RPE = rating of perceived exertion.

**Table 2:** Reliability data for MVIC torque, EMG AMP, and NME during the pre-test forearm flexion MVICs at RPE = 2 and RPE = 8.

Variables (mean ± SD)	Visit 1	Visit 2	*P*	ICC	ICC_95%_
MVIC (Nm)	78.1 ± 19.6	80.4 ± 19.4	0.379	0.902	0.707–0.970
EMG AMP (μVrms)	1123.2 ± 626.0	1325.6 ± 712.5	0.020 *	0.892	0.523–0.971
NME	0.09 ± 0.05	0.08 ± 0.05	0.088	0.903	0.681–0.972

*p*= Alpha from the ANOVA for systematic error; ICC = intraclass correlation coefficient; ICC_95%_ = ICC 95% confidence interval; MVIC = maximal voluntary isometric contraction; EMG AMP = electromyographic amplitude; NME = neuromuscular efficiency. * Significant mean difference from Visit 1 to Visit 2.

**Table 3:** Polynomial regression models, correlations (Corr.), and *p*-values for normalized torque, EMG AMP, and NME vs. time relationships during the sustained, isometric forearm flexion task anchored to RPE = 2 (RPE2_FT_) at an elbow joint angle of 100°.

Subjects	Torque			EMG AMP			NME		
	Model	Corr.	*p*-Value	Model	Corr.	*p*-Value	Model	Corr.	*p*-Value
1	Linear	−0.770	0.009	-	-	NS	Linear	−0.708	0.033
2	Linear	−0.949	<0.001	Linear	−0.792	0.006	-	-	NS
3	Linear	−0.953	<0.001	-	-	NS	Linear	−0.923	<0.001
4	Linear	−0.861	0.001	Linear	−0.807	0.005	Linear	−0.940	<0.001
5	Linear	−0.956	<0.001	Linear	−0.930	<0.001	Linear	−0.704	0.034
6	Linear	−0.978	<0.001	Linear	−0.885	<0.001	Linear	−0.955	<0.001
7	Linear	−0.967	<0.001	Linear	−0.756	0.011	Linear	−0.817	0.007
8	Linear	−0.799	0.006	-	-	NS	-	-	NS
9	Linear	−0.897	<0.001	-	-	NS	Linear	−0.764	0.016
10	Linear	−0.956	<0.001	Linear	−0.848	0.002	Linear	−0.953	<0.001
11	Linear	−0.836	0.003	-	-	NS	Linear	−0.916	<0.001
12	Linear	−0.855	0.002	Linear	−0.723	0.018	Linear	−0.921	<0.001
Composite	Linear	−0.958	<0.001	Linear	−0.776	0.008	Linear	−0.976	<0.001

NS = not significant.

**Table 4:** Polynomial regression models, correlations (Corr.), and *p*-values for normalized torque, EMG AMP, and NME vs. time relationships during the sustained, isometric forearm flexion task anchored to RPE = 8 (RPE8_FT_) at an elbow joint angle of 100°.

Subjects	Torque			EMG AMP			NME		
	Model	Corr.	*p*-Value	Model	Corr.	*p*-Value	Model	Corr.	*p*-Value
1	Linear	−0.872	<0.001	Linear	−0.635	0.049	-	-	NS
2	Linear	−0.908	<0.001	Linear	−0.947	<0.001	Linear	−0.865	0.003
3	Quadratic	−0.969	0.021	Linear	−0.635	0.048	Linear	−0.699	0.036
4	Quadratic	−0.959	0.024	Quadratic	−0.88	0.012	-	-	NS
5	Linear	−0.933	<0.001	Linear	−0.714	0.02	Quadratic	−0.408	0.005
6	Linear	−0.761	0.011	Linear	−0.731	0.016	-	-	NS
7	Linear	−0.947	<0.001	-	-	NS	Linear	−0.799	0.010
8	Linear	−0.884	<0.001	-	-	NS	-	-	NS
9	Linear	−0.905	<0.001	-	-	NS	Linear	−0.716	0.030
10	Linear	−0.933	<0.001	Quadratic	−0.436	0.031	Linear	−0.940	<0.001
11	Linear	−0.944	<0.001	-	-	NS	Linear	−0.831	0.006
12	Quadratic	−0.993	0.003	Quadratic	−0.476	0.007	Linear	−0.946	<0.001
Composite	Linear	−0.969	<0.001	Linear	−0.857	0.002	Linear	−0.969	<0.001

NS = not significant.

**Table 5:** Spearman’s rank order correlations (r_s_) for the time to task failure (TTF) values and performance fatigability (PF = % change in maximal torque from pre-test to post-test MVIC assessments) from the sustained, isometric forearm flexion task anchored to RPE = 2 (RPE2_FT_) and the 10 Likert-type items from the post-test questionnaire provided after the RPE2_FT_.

	BB Fatigue	BB Pain	FM Fatigue	FM Pain	HM Fatigue	HM Pain	LOF	Motivation 1	Motivation 2	Motivation 3	TTF	PF
BB Fatigue	1.000											
BB Pain	0.663 *	1.000										
FM Fatigue	0.694 *	0.539	1.000									
FM Pain	0.497	0.675 *	0.788 **	1.000								
HM Fatigue	0.654 *	0.720 **	0.763 **	0.689 *	1.000							
HM Pain	0.520	0.648 *	0.617 *	0.585 *	0.942 **	1.000						
LOF	0.294	0.287	0.241	0.449	−0.017	−0.120	1.000					
Motivation 1	0.210	−0.027	0.172	0.000	0.309	0.242	−0.442	1.000				
Motivation 2	−0.186	0.033	0.085	0.233	−0.092	−0.204	0.703 *	−0.308	1.000			
Motivation 3	−0.173	−0.216	0.106	0.155	−0.360	−0.443	0.691 *	−0.311	0.822 *	1.000		
TTF	−0.074	0.249	0.167	0.458	0.253	0.306	−0.167	0.159	−0.015	0.017	1.000	
PF	0.315	−0.256	0.278	0.137	−0.121	−0.263	0.289	0.397	−0.015	0.309	−0.210	1.000

Regarding contribution to the decision to terminate the task, the 10 Likert-type items (biceps brachii fatigue, biceps brachii pain, forearm muscles fatigue, forearm muscles pain, hand muscles fatigue, hand muscles pain, loss of focus, Motivation 1, Motivation 2, and Motivation 3) were rated on a 5-point (1–5) Likert-type scale with definitions associated with the following numbers: 1 = strongly disagree; 2 = disagree; 3 = neither agree nor disagree; 4 = agree; 5 = strongly agree. MVIC = maximal voluntary isometric contraction; BB = biceps brachii; FM = forearm muscles; HM = hand muscles; PF = [((pre-test MVIC − post-test MVIC)/pre-test MVIC) × 100]; LOF = loss of focus. * *p* ≤ 0.05, r_s_ = 0.584; ** *p* ≤ 0.01, r_s_ = 0.724.

**Table 6:** Spearman’s rank order correlations (r_s_) for the time to task failure (TTF) values and performance fatigability (PF = % change in maximal torque from pre-test to post-test MVIC assessments) from the sustained, isometric forearm flexion task anchored to RPE = 8 (RPE8_FT_) and the 10 Likert-type items from the post-test questionnaire provided after the RPE8_FT_.

	BB Fatigue	BB Pain	FM Fatigue	FM Pain	HM Fatigue	HM Pain	LOF	Motivation 1	Motivation 2	Motivation 3	TTF	PF
BB Fatigue	1.000											
BB Pain	0.518	1.000										
FM Fatigue	0.051	0.302	1.000									
FM Pain	−0.008	0.157	0.904 **	1.000								
HM Fatigue	0.142	0.482	0.726 **	0.630 *	1.000							
HM Pain	0.222	0.442	0.679 *	0.632 *	0.856 **	1.000						
LOF	0.170	−0.125	0.238	0.496	0.175	0.377	1.000					
Motivation 1	0.064	0.204	0.408	0.184	0.310	0.008	−0.219	1.000				
Motivation 2	0.343	0.129	0.437	0.565	0.077	0.329	0.690 *	−0.026	1.000			
Motivation 3	−0.419	−0.274	0.272	0.611 *	0.000	0.134	0.548	−0.309	0.378	1.000		
TTF	−0.158	−0.137	−0.092	−0.165	−0.223	−0.166	−0.315	−0.148	−0.220	0.000	1.000	
PF	0.415	0.547	0.392	0.319	0.037	0.090	−0.158	0.070	0.318	0.065	0.042	1.000

Regarding contribution to the decision to terminate the task, the 10 Likert-type items (biceps brachii fatigue, Biceps brachii pain, forearm muscles fatigue, forearm muscles pain, hand muscles fatigue, hand muscles pain, loss of focus, Motivation 1, Motivation 2, and Motivation 3) were rated on a 5-point (1–5) Likert-type scale with definitions associated with the following numbers: 1 = strongly disagree; 2 = disagree; 3 = neither agree nor disagree; 4 = agree; 5 = strongly agree. MVIC = maximal voluntary isometric contraction; BB = biceps brachii; FM = forearm muscles; HM = hand muscles; PF = [((pre-test MVIC − post-test MVIC)/pre-test MVIC) × 100]; LOF = loss of focus. * *p* ≤ 0.05, r_s_ = 0.584; ** *p* ≤ 0.01, r_s_ = 0.724.

**Table 7:** Individual subject responses for each item from the post-test questionnaire following the sustained, isometric forearm flexion task anchored to RPE = 2 (RPE2_FT_).

Subjects	RPE2_FT_									
	BB Fatigue	BB Pain	FM Fatigue	FM Pain	HM Fatigue	HM Pain	LOF	Motivation 1	Motivation 2	Motivation 3
1	2	2	4	4	5	5	1	5	1	1
2	5	2	2	2	4	3	2	5	1	1
3	1	2	1	2	4	4	2	3	2	1
4	1	1	1	1	1	1	2	4	3	3
5	2	1	2	1	4	4	1	4	1	1
6	4	3	3	2	5	4	2	4	2	1
7	2	1	2	2	2	2	4	3	2	2
8	2	2	4	4	4	3	3	3	3	3
9	1	1	1	1	1	1	1	5	1	1
10	2	2	2	2	2	2	4	2	2	2
11	4	4	2	2	4	4	2	3	1	1
12	1	1	1	1	1	1	1	1	1	1
Mean ± SD	2.3 ± 1.4	1.8 ± 0.9	2.1 ± 1.1	2.0 ± 1.0	3.1 ± 1.6	2.8 ± 1.4	2.1 ± 1.1	3.5 ± 1.2	1.7 ± 0.8	1.5 ± 0.8

Regarding contribution to the decision to terminate the task, the 10 Likert-type items (biceps brachii fatigue, biceps brachii pain, forearm muscles fatigue, forearm muscles pain, hand muscles fatigue, hand muscles pain, loss of focus, Motivation 1, Motivation 2, and Motivation 3) were rated on a 5-point (1–5) Likert-type scale with definitions associated with the following numbers: 1 = strongly disagree; 2 = disagree; 3 = neither agree nor disagree; 4 = agree; 5 = strongly agree. BB = biceps brachii; FM = forearm muscles; HM = hand muscles; LOF = loss of focus.

**Table 8:** Individual subject responses for each item from the post-test questionnaire following the sustained, isometric forearm flexion task anchored to RPE = 8 (RPE8_FT_).

Subjects	RPE8_FT_									
	BB Fatigue	BB Pain	FM Fatigue	FM Pain	HM Fatigue	HM Pain	LOF	Motivation 1	Motivation 2	Motivation 3
1	4	4	4	3	4	3	1	5	1	1
2	5	4	2	2	1	1	1	4	2	1
3	4	5	4	3	4	4	1	5	2	1
4	5	4	2	2	3	3	3	4	2	1
5	4	5	2	2	4	2	1	5	1	1
6	5	4	4	4	5	4	2	5	2	1
7	4	4	3	4	2	2	2	4	2	2
8	2	3	3	4	4	4	3	3	2	2
9	4	2	1	1	1	1	1	5	1	1
10	5	5	2	2	3	3	2	4	2	1
11	5	5	2	2	4	4	1	1	1	1
12	1	1	1	1	1	1	1	1	1	1
Mean ± SD	4.0 ± 1.3	3.8 ± 1.3	2.5 ± 1.1	2.5 ± 1.1	3.0 ± 1.4	2.7 ± 1.2	1.6 ± 0.8	3.8 ± 1.5	1.6 ± 0.5	1.2 ± 0.4

Regarding contribution to the decision to terminate the task, the 10 Likert-type items (biceps brachii fatigue, biceps brachii pain, forearm muscles fatigue, forearm muscles pain, hand muscles fatigue, hand muscles pain, loss of focus, Motivation 1, Motivation 2, and Motivation 3) were rated on a 5-point (1–5) Likert-type scale with definitions associated with the following numbers: 1 = strongly disagree; 2 = disagree; 3 = neither agree nor disagree; 4 = agree; 5 = strongly agree. BB = biceps brachii; FM = forearm muscles; HM = hand muscles; LOF = loss of focus.

**Table 9:** Composite responses for the 10 Likert-type items from the post-test questionnaire provided after the sustained, isometric forearm flexion tasks anchored to RPE = 2 (RPE2_FT_) and RPE = 8 (RPE8_FT_), respectively.

Sustained Task	Likert-Type Scale Ratings	Percentage (% of Sample)									
		BB Fatigue	BB Pain	FM Fatigue	FM Pain	HM Fatigue	HM Pain	LOF	Motivation 1	Motivation 2	Motivation 3
RPE2_FT_	1—Strongly disagree	33	42	33	33	25	25	33	8	50	67
	2—Disagree	42	42	42	50	17	17	42	8	33	17
	3—Neither agree or disagree	0	8	8	0	0	17	8	33	17	17
	4—Agree	17	8	17	17	42	33	17	25	0	0
	5—Strongly agree	8	0	0	0	17	8	0	25	0	0
RPE8_FT_	1—Strongly disagree	8	8	17	17	25	25	58	17	42	83
	2—Disagree	8	8	42	42	8	17	25	0	58	17
	3—Neither agree or disagree	0	8	17	17	17	25	17	8	0	0
	4—Agree	42	42	25	25	42	33	0	33	0	0
	5—Strongly agree	42	33	0	0	8	0	0	42	0	0

BB = biceps brachii; FM = forearm muscles; HM = hand muscles; LOF = loss of focus.

## Data Availability

The data sets generated during and/or analyzed during the present study are available from the corresponding author upon reasonable request.
